# Exploring challenges related to health literacy of Albanian immigrants in Germany: a qualitative study among a younger, educated cohort

**DOI:** 10.1186/s12913-026-15939-4

**Published:** 2026-11-05

**Authors:** Filip Pistulli, Christian Keinki, Peter Noack, Katharina Eckstein, Jutta Hübner, Emadaldin Ahmadi

**Affiliations:** 1 Klink für Innere Medizin II, Universitätsklinikum Jena, Am Klinikum 1, 07747 Jena, Germany; 2 Lehrstuhl für pädagogische Psychologie, Humboldtstr. 27, 07743 Jena, Germany

**Keywords:** Immigration, Health literacy, Albanian immigrants, Integration, Qualitative research, Healthcare system, Germany

## Abstract

**Background:**

Albanian immigrants in Germany face health literacy challenges due to language barriers, cultural differences, and unfamiliarity with the healthcare system, which intersect with systemic issues such as medical staff shortages.

**Purpose:**

This qualitative study explored the health literacy and health challenges of Albanian immigrants in Germany, examining their capacity to navigate healthcare services, make informed decisions, and identify barriers and facilitators influencing their experiences.

**Methods:**

Semi-structured interviews were conducted with 15 Albanian immigrants (mean age 27; predominantly younger and highly educated). Participants were recruited via online communities and cultural centers. Data were analyzed using thematic content analysis guided by thematic saturation principles.

**Results:**

Participants reported multiple barriers to healthcare access. Language proficiency gaps impeded accurate communication of medical concerns, with several describing difficulty understanding diagnoses, treatment plans, and medical terminology. Bureaucratic delays and long waiting times for appointments deterred timely care utilization, and securing a family doctor proved challenging depending on region of residence. Despite these obstacles, participants expressed strong trust in physicians and satisfaction with treatment quality once received. Statutory health insurance mitigated financial concerns for most. Systemic strains, particularly staff shortages, exacerbated communication gaps and care delays, with physician participants acknowledging workforce deficits contribute to prolonged waiting periods. Shared decision-making was inconsistently practiced; some reported needing to insist on referrals. Experiences of friends and relatives further shaped expectations, with accounts of misdiagnosis due to language barriers reinforcing concerns about systemic responsiveness.

**Conclusions:**

Socioeconomic factors, including education level, employment status, and insurance coverage, shaped participants’ healthcare experiences, with higher education and statutory insurance mitigating financial concerns, yet systemic barriers such as bureaucratic delays, communication gaps and provider shortages persisted regardless of socioeconomic position. Despite these barriers, participants maintained trust in the system, valuing statutory health insurance, quality treatment, and provider professionalism.

**Supplementary information:**

The online version contains supplementary material available at https://doi.org/10.1186/s12913-026-15939-4.

## Introduction

Millions of people have been emigrating from their native nations [1] (1990–2017), in pursuit of better job prospects and a better quality of life.

Among these groups, the Albanian diaspora has emerged as a significant community contributing to the cultural and demographic shifts in host countries. The number of Albanian immigrants in Germany has steadily increased over the years, forming a notable population whose unique challenges and contributions merit closer examination [1, 2]. Integration is essential for immigrants to adapt fully to the customs, rules, and societal frameworks of their host country while effectively addressing barriers such as language and cultural differences [3].

Beyond individual challenges, structural and systemic factors significantly shape migrant health outcomes. These include institutional racism, employment discrimination, bureaucratic hurdles in accessing social services, and unequal distribution of healthcare resources [2, 4–7]. Employment barriers, such as precarious work or unrecognized qualifications, often limit immigrants’ eligibility for comprehensive health insurance, exacerbating socioeconomic disparities [4, 8, 9]. Discrimination in healthcare settings, including cultural insensitivity and language-related dismissals, further erodes trust in providers, discouraging timely medical consultations [5, 10]. Bureaucratic challenges, such as residency permit requirements and fragmented eligibility criteria, disproportionately burden marginalized groups, worsening health inequities [2, 4, 6].

In this study, the term “healthcare” (one word) is deliberately standardized to reflect the interconnected systems, policies, and practices that shape immigrant health experiences in Germany. This choice aligns with contemporary academic and clinical trends prioritizing the term to describe holistic systems rather than isolated acts of care [11].

One of the critical facets of successful integration is health literacy, a key determinant of health equity, safety, and quality of care [8, 12–15]. Health literacy is defined as an individual’s ability to access, understand, and use information to make informed decisions about their health [16]. However, immigrant communities, including Albanians in Germany, often encounter significant obstacles in developing and sustaining adequate health literacy [17]. These challenges are exacerbated by language barriers, cultural differences, and unfamiliarity with the structure and procedures of the host country’s healthcare system.

Within the German context, systemic strains such as medical staff shortages and fragmented care delivery disproportionately affect marginalized groups, including immigrants [18–22]. By 2025, Germany’s projected deficit of 20,000 junior general practitioners will exacerbate appointment delays and reduce consultation time, particularly impacting immigrants who already face linguistic and cultural barriers [18, 19]. Fragmented care, exemplified by restrictive policies for asylum seekers (e.g., limited access to non-acute treatments and reliance on vouchers), delays critical care and worsens health outcomes [2, 4]. For Albanian immigrants, these systemic challenges intersect with cultural and linguistic barriers, creating compounded obstacles to health literacy and equitable care. Studies highlight that limited German proficiency impedes interpretation of health information and communication with providers, a gap amplified by understaffed clinics and long wait times [14, 20, 21, 23].

The purpose of this study is to investigate the health literacy of Albanian immigrants in Germany, a population underrepresented in public health research despite its growing presence, unique socio-cultural context, and distinct migration patterns [1, 2], by exploring the extent to which they understand, access, and utilize healthcare services, examining their capacity to make informed health decisions, identifying the barriers and facilitators that influence their experiences, and situating these findings within the broader discourse on immigrant health at the intersection of health literacy, integration, and systemic healthcare challenges.

## Methods

### General design

In this study, a qualitative data-gathering method was utilized using semi-structured interviews, conducted with Albanian immigrants. Specific content analysis followed data-gathering. Participants who self-identified as Albanian are individuals who speak Albanian in their mother language, and whose origin comes from Albanian-speaking regions which can go beyond the border of where Albania is located. We adhered to the reporting guideline for qualitative research provided by SRQR.

### Participants

The study population comprised of 15 individuals aged 18 years or older, who self-identify as Albanian and residing in Germany at the time of the Interview which included healthcare professionals, students, and workers in different fields such as economics and business. Participants were recruited using a combination of purposive and snowball sampling strategies to ensure representation of diverse experiences while maintaining focus on the research objectives.

Inclusion criteria required individuals to: (1) self-identify as Albanian (native Albanian speakers from Albanian-speaking regions), (2) be aged ≥ 18 years, and (3) have experiences with German healthcare. Individuals were excluded from participation if they were (1) under 18 years of age, (2) were unable to provide informed consent due to cognitive impairment, severe mental illness, or language barriers unrelated to Albanian proficiency, (3) had no experience accessing or attempting to access German healthcare services, or (4) were Albanian speakers from Albanian-speaking regions who did not self-identify as Albanian due to political or ethnic identification with other groups.

For our purposive sampling we sought diversity in age, gender, education, occupation, and length of stay. Recruitment posts were shared in Albanian-focused Facebook groups, Instagram, and WhatsApp communities.

We employed snowball sampling guided by the principle of thematic saturation [24, 25]. After interviews, participants were asked to refer other Albanian immigrants from their networks who met inclusion criteria. This helped reach individuals less active on social media or belonging to less visible subgroups. Referrals were included only if they met purposive criteria, and sample composition was monitored to prevent over-representation of any subgroup. Interested individuals from social media posts were screened for eligibility. Following each interview, participants received researcher contact information to share with potential referrals. This hybrid approach leveraged community networks while maintaining analytical focus.

### Researcher characteristics and credentials

The research team comprised individuals with diverse disciplinary expertise to ensure comprehensive analysis. The first author (F.P.) is a medical student with training in qualitative and quantitative research and native Albanian language proficiency. Dr. Christian Keinki (C.K.) and Dr. Emadaldin Ahmadi (E.A.) are healthcare researchers at the University Clinic Jena with extensive experience in oncology, health services research, and qualitative methodology. Prof. Dr. Jutta Hübner (J.H.) is a professor of integrative oncology with expertise in patient communication and health literacy. Prof. Peter Noack (P.N.) and Dr. Katharina Eckstein (K.E.) are psychologists specializing in educational psychology, migration, and adolescent development, contributing expertise on integration and sociocultural adaptation. This multidisciplinary composition allowed for balanced interpretation of findings from medical, psychological, and sociocultural perspectives.

### Data collection

The data-gathering consisted of open-ended questions and was used in semi-structured interviews conducted in a private setting. The open questions are formulated in such a way that they offer the participants the greatest possible flexibility and freedom in their answers. Participants were asked for their informed consent prior to the interviews. Voice recording and note-taking were used during the interviews, which were planned with the study’s objectives in mind.

The interviews lasted from 10 to 30 minutes. Interviews were conducted in Albanian by one interviewer and were subsequently translated into English for analysis. After gathering information on demographic data, the questions from the guideline (Additional files 1 and 2) were used for the interview, which consisted of four parts with the following topics:

1. Obtaining information on health information and services, knowledge about the functioning of the healthcare system in Germany.
2. Barriers to accessing the healthcare system and using healthcare services.
3. Past experiences with the healthcare system in relation to health/illness.
4. Expectations on healthcare system/healthcare workers.

### Data analysis

Following the completion of each interview, two researchers independently analyzed the transcripts using reflexive thematic analysis [26], a flexible and interpretative approach suited for exploring participants’ lived experiences and sociocultural contexts. This method aligns with our epistemological stance, emphasizing co-construction of meaning between researchers and participants [27]. The six-phase process included:

- Familiarization with data: Researchers immersed themselves in the data by reading and re-reading translated transcripts (interviews were conducted in Albanian and translated into English for analysis)
- Generating initial codes: Codes were inductively derived from participants’ narratives, focusing on explicit and latent meanings.
- Searching for themes: Codes were clustered into preliminary themes (e.g., “language barriers,” “trust in providers”) through collaborative discussions.
- Reviewing themes: Themes were refined by revisiting coded extracts and the entire dataset to ensure coherence and relevance.
- Defining/naming themes: Each theme was contextualized within participants’ sociocultural experiences (e.g., “Bureaucratic Delays as Systemic Marginalization”).
- Producing the report: Findings were synthesized into a narrative that foregrounded participants’ voices while critically reflecting on researchers’ interpretive roles.

To enhance scientific rigor, we attended to reflexivity throughout the research process. The first author (F.P.), who conducted all interviews, shares Albanian ethnicity and native language proficiency with participants, facilitating trust and cultural understanding. However, this insider status, combined with F.P.‘s background as a medical student, may have shaped data collection and interpretation. To mitigate potential bias, the research team engaged in regular reflexive discussions, maintained memos documenting analytical decisions, and employed member checking [28] within a time frame of 1–2 months to ensure interpretations aligned with participants’ experiences. Coding discrepancies (e.g., differing interpretations of “cultural differences”) were resolved through iterative discussions that explicitly considered alternative perspectives. Participants were provided with a summary of the findings and invited to offer feedback on the interpretations to confirm that the results accurately reflected their experiences and perspectives.

Initial interviews provided valuable insights and helped refine the interview guide. As the study progressed, the researcher observed patterns and commonalities in the responses, noting when new information ceased to emerge.

The decision to conclude the participant recruitment phase was informed by the principle of thematic saturation. It became evident during the concurrent analysis that the interviews were consistently yielding similar themes and perspectives, and no novel codes or themes were surfacing. The saturation point was reached after 15 participants when successive interviews failed to generate new inductive themes or additional depth to the existing thematic framework.

### Ethical considerations

The ethics approval was approved by the Ethical Committee of University Clinic Jena (UKJ) (Reg. No. 2022–2856-Bef). Before participation, all individuals were informed that participation was voluntary, and all gave written informed consent. All procedures performed in this study were in accordance with the ethical standards of the institutional research committee and with the 1964 Helsinki Declaration and its later amendments.

## Results

Altogether there were 15 participants for the qualitative interviews, 5 of which were female and 10 were male with a mean age of 27 (see Table 1).

**Table 1:** Demographic characteristics of the subjects

Participant Code	Gender	Age range	Education	Religion	Languages spoken	Household composition	Existing chronic diseases	Current work status
1	Male	20–30	High school, currently in university	Prefers not to answer	Albanian native, English C2, Spanish B2, German B1	Lives with family	Recurrent minor condition	Student, studying architecture, working part time in the field
2	Female	20–30	High school, currently studying at university	Atheist	Albanian native, English C2, German B2	Lives with family	Recurrent minor conditions	Student
3	Male	20–30	High school, currently in university	Religious, no further information was provided	Albanian native, English C2, Russian B2, German B1, Bulgarian B1	Lives alone	No chronic illnesses	Linguistic student, freelance translator, language teacher
4	Female	20–30	Bachelor	Christian	Albanian native, English native, Italian fluent, Spanish fluent, Chinese fluent, French intermediate, German beginner	Lives with roommate	Chronic condition (managed)	Student
5	Female	20–30	Bachelor	Catholic	Albanian native, English advanced, Chinese advanced, German intermediate, Spanish intermediate	Lives alone	Chronic condition (managed)	Marketing advisor
6	Male	20–30	High school	Islam	Albanian native, English B2, German A2	Lives with sibling	Minor hereditary condition	Student with part time work
7	Male	50–60	Post graduate	Christian	Albanian native, German C1, English B1	Lives with spouse	Chronic condition (managed)	Physician
8	Male	30–40	Medical faculty, university	Islam	Albanian native, English C1, German C1, Spanish B1	Lives with spouse	No chronic illnesses	Physician
9	Male	20–30	Master on economics	Religious, no further information was provided	Albanian native, English C1, German B2, Turkish B1, Italian A2	Lives alone	No chronic illnesses	Manager on human resources and finance specialist
10	Male	20–30	Medical university	Islam	Albanian native, English C1, German C1, Serbian A1	Lives alone	Mild mental health condition (resolved)	Physician
11	Male	20–30	Medical university	Islam	Albanian native, English C1, German B1	Lives with spouse	No chronic illnesses	Researcher in a laboratory
12	Female	50–60	University of Tirana, economy	Religious, no further information was provided	Albanian native	Lives with family	No chronic illnesses (family members have conditions)	Economist
13	Female	20–30	Bachelor	Atheist	Albanian native, English C1, German C1, Spanish A2, Italian A2, Turkish A2,	Lives alone	No chronic illnesses	Student
14	Male	27	Master on economics	Agnostic	Albanian native, English C1, German B2, Italian B2, Turkish B1	Lives with fiancé	No chronic illnesses	Business manager
15	Male	20–30	Master on economics	Atheist	Albanian native, English C1, German B2, French A2	Lives alone	No chronic illnesses	Researcher

Content analysis included 4 themes regarding different aspects on health literacy, including information gathering on the side of the participants, barriers to accessing healthcare, past experiences and their expectation respectively. Each theme contained from 3 to 5 subthemes to further explore the many aspects residing in each topic. All the sentences in quotation marks are from participants.

### Obtaining information on health information and services, knowledge about the functioning of the healthcare system in Germany

#### Resources for information about health, illness and treatment

Most participants seem to be able to receive some information from the family doctor (Hausarzt/in) or specialized doctors (Facharzt/in), some of them seem to get this information through friends and family. However, there is still a substantial amount of people who do not seem to possess sufficient information regarding this topic. Most of them seem to use the internet to fill the knowledge gap regarding health services.

*I have used the internet before for more details on specific situations such as for the COVID-19 treatment.* (Participant code #1)

#### Trust regarding health issues

Another aspect on health literacy is the person that immigrants trust the most regarding their health. This is relevant to establish because to receive proper help, people in need of it have to make an effort to get it from established and recognized pillars of health. The answer to this question was substantially the same. All but one, who had more trust in alternative than conventional medicine, agreed that the physician is the person they trust the most regarding health.

*Of course, when it comes to receiving the best medical treatment, I would listen to the doctor in charge. In case of more specific illnesses, I would listen to what the specialized physician had to say.* (Participant code #5)

#### The investigation of proper healthcare use for adequate management of the health issue

This subtheme examined immigrants’ capacity to navigate healthcare services effectively based on their specific medical needs. To elucidate this capability, participants were presented with two distinct clinical scenarios: acute illness requiring immediate intervention and chronic conditions necessitating long-term care management. This methodological approach facilitated a comparative analysis of healthcare-seeking behaviors across different medical contexts. 13 of the participants expressed that the visit to the emergency would happen in case of an acute illness with the suspicion of something severe, such as immediate heavy pains or chest pains of an acute nature. 12 of them admitted, in case of an illness that does on not require immediate treatment the first person they would visit is the family doctor. If further treatment was required, then the visit to the specialized physician would follow.

*For acute problems, I would go to the emergency room. The doctor is the most important for to get the best treatment. If it is something that can wait, then I would go to the family doctor.* (Participant code #7)

### Barriers to accessing the healthcare system and using healthcare services

To fully articulate the challenges faced by immigrants of Albanian descent, it was necessary to examine the barriers preventing this population from accessing adequate healthcare. This theme showcased many answers, suggestions and complaints. The following subthemes were created for the investigation of this purpose.

#### Barriers preventing access to healthcare services

Like many immigrant populations, Albanian immigrants face significant language barriers that impede access to adequate healthcare. The ability to communicate effectively with healthcare professionals remained a critical factor in ensuring proper diagnosis and treatment. Several participants with strong English proficiency noted—during a follow-up inquiry—that physicians who communicated effectively in English significantly facilitated the accurate transmission of medical information, eliminating reliance on gestures or nonverbal cues. Additionally, immigrants who had resided in Germany for an extended period or possessed German language skills encountered fewer such barriers.

*The German language for example it’s difficult to understand, especially when it comes to the medical terminology to get the point across. Sometimes it feels that the doctor hasn’t fully understood my issues.* (Participant code #6)

Ten subjects mentioned as a very important factor the bureaucracy and waiting hours in health institutions and facilities. When asked to disclose further information some subjects expressed the following:

*The bureaucracy and general procedures. The test and analyses take too long and it’s almost too much time wasted.* (Participant code #13)

Several others explained that even the finding of a family doctor willing to take them in for clinical examination as new patients could be very difficult depending on the region of residency (follow-up inquiry).

#### Evaluation of healthcare services

Participants expressed dissatisfaction with procedural inefficiencies and excessive appointment delays. Several reported instances of requiring appointments to be scheduled months in advance, a factor that further deterred timely healthcare utilization.

*Sometimes the availability is lacking. Sometimes I would rather not bother reaching to receive a medical service because I think I would not receive it at the right time*.

(Participant code #12)

The majority of the participants however, expressed satisfaction with the treatment they received and did not have many complaints regarding the issues mentioned above.

#### Trust, regarding illness and its treatment

This subtheme aimed to assess whether participants perceived Germany’s established medical institutions and facilities as appropriate for treatment. Consistent with the preceding question regarding trust in healthcare providers, participants’ responses demonstrated minimal variation. 12 of them admitted that the doctor was the most important figure. When asked to further elaborate, 7 expressed the importance of a specialized doctor, with many years of experience. A number of several participants mentioned that the hospital center and medical staff that worked together as a team, not only including individual experienced doctors, played an important role.

*Medicine in general, the hospital and how good the specialized doctors are.* (Participant code #10)

#### Languages and cultural differences

The first subtheme addressed multiple concerns arising from the open-ended questions, revealing divergent opinions among participants. Four participants expressed minimal concerns regarding the language. Four others showcased confidence in the language proficiency. Six showed severe concerns due to individual health status and language proficiency problems. None of subjects expressed major distress regarding cultural differences. While general language barriers were acknowledged, medical terminology posed a distinct challenge, complicating both communication with providers and comprehension of health information. Participants with severe concerns and limited German proficiency reported difficulties understanding diagnoses, treatment plans, and medication instructions, often leading to misunderstandings or incomplete adherence to care.

*The doctor used words like ‘Überweisung’ and ‘antihypertensive’ during my appointment. I nodded to avoid embarrassment, but later I had to Google-translate the terms to understand what they meant. I find it difficult to express myself in German fluently to accurately convey my medical problems. Often I feel like the doctor doesn’t understand me. (Participant code #5)*

This aligned with studies highlighting that medical jargon disproportionately affects non-native speakers, who may lack exposure to technical terms even if conversational German is adequate [23, 29].

#### Financial concerns in case of serious illness

A substantial number of participants expressed low level of concern regarding serious illness. Germany’s statutory health insurance system covers the majority of healthcare costs for insured patients facing severe illness. When combined with adequate knowledge of the German healthcare system (for the reasons previously outlined) and participants’ personal medical histories, this coverage provides substantial protection against financial hardship resulting from serious health conditions. Subjects who also mentioned that they were not in possession of a statutory health insurance or of medical issues which they know that are not covered from statutory health insurance such as dental health problems, expressed more distress on the matter, including subjects who lacked knowledge in the coverage range of the statutory health insurance and health insurance policies in Germany generally.

*Yes, I am but to a small degree. It also depends on the illness. If it isn’t covered by insurance, I would be worried. But most of illnesses are covered by insurances here in Germany.* (Participant code #15)

### Past experiences with the healthcare system in relation to health/illness

A detailed exploration of past medical experiences in Germany of the study group was considered necessary. With the help of the subthemes below, information regarding past medical issues and their respective treatment or lack thereof was gathered and evaluated.

#### Mental and physical health problems and the association of the healthcare system regarding treatment

On this subtheme, information was gathered concerning previous existing health concerns of the participants and the part of the healthcare that the subjects tried to reach out to.

It consisted of past medical experiences and treatments among participants, shedding light on their interactions with the German healthcare system. It demonstrated a diverse range of health issues encountered, including gastrointestinal problems, musculoskeletal conditions, mental health concerns, and general illnesses like flu (Additional file 3).

Participants sought treatment from various healthcare providers, including family doctors, specialists, hospitals, and emergency departments. Some faced challenges such as bureaucratic delays or difficulties in accessing specialized care due to lack of a family doctor. Bureaucratic delays in Germany’s healthcare system disproportionately affected Albanian immigrants due to complex insurance enrollment (e.g., missing documents, language barriers) and gatekeeping referral requirements that prolonged services from specialized physicians. Asylum seekers (such as Participant code #12) encountered additional barriers under restrictive policies, delaying non-emergency treatments. Administrative inefficiencies, such as lost paperwork, worsened delays, while cultural mismatches with Germany’s referral system bred distrust.

Understanding these experiences was crucial for the research as it provided insights into the participants’ interactions with the healthcare system, highlighting areas where interventions or support may be needed to improve health outcomes and access to healthcare for Albanian immigrants and refugees in Germany.

#### Level of satisfaction regarding treatment

The majority of participants reported positive evaluations of the medical treatment they received. Particular satisfaction was expressed regarding outcomes for condition-specific interventions. The following statements exemplify participant feedback reflecting treatment satisfaction:

*Yes, because after only 5 days I could see significant improvements of my symptoms and stomach aches.* (Participant code #7)

*Yes, it was. I felt that the service was quite good in both cases, since I saw significant improvement in comparison to my condition before the treatment.* (Participant code #15)

This analysis focuses exclusively on patient satisfaction with direct physician and clinical staff interventions. Systemic aspects of treatment within the broader healthcare framework are examined subsequently in dedicated subthemes.

However, there were participants who felt that the treatment was insufficient. Below such a statement is mentioned.

*We went to the emergency department and we got supplied with medication regarding these the illnesses mentioned above. However, it felt that it was insufficient because still no treatment was given regarding exostosis.* (Participant code #12)

#### Satisfaction with the medical treatment received and shared decision making

While participants expressed general satisfaction with treatment outcomes, shared decision making was inconsistently practiced, particularly for patients with chronic or complex conditions. Participants reported that providers often prioritized efficiency over collaboration, leading to insufficient explanations of treatment options. For example, the following was noted:

The family doctor prescribed me low-effect medication … It was only after severely insisting, that I got the treatment that I wanted. (the referral for an MRI)

(Participant #14)

This aligns with Participant #12’s experience, where their children’s chronic conditions (exostosis and epilepsy) were inadequately addressed due to providers’ reluctance to explore alternative treatments. Such dismissiveness reflects a systemic undervaluing of patient agency, particularly for immigrants perceived as less knowledgeable about healthcare norms

### Expectations on healthcare system/healthcare workers

#### Expectations on medical staff

This subtheme examined the hypothetical scenario of a healthcare system comprehensively addressing all patient needs according to clinical profiles. The responses exhibited variability, with predominant themes revolving around issues such as waiting times and the conduct of the medical staff. These themes encompassed aspects such as competence, willingness to listen, empathy, the necessity for a detailed explanation during examination, provision of information on treatment and medication plans even in the absence of direct patient inquiries and allocating sufficient time for comprehensive information dissemination. A lot of value was put into regarding the patient in all aspects of healthcare including physical and mental needs. The nurses were also encouraged to contribute on the informing of the patient in case of a stationary stay of the patient. Some participants reported experiencing prejudice rooted in racial and cultural biases, a phenomenon well-documented in European healthcare settings [30].

*I would first and foremost like to receive the best treatment for my illness. The doctor needs to be communicative. Different treatment options. Hearing until the end about what the patient has to say regarding his physical and mental health.* (Participant code #4)

#### Satisfaction in the use of the healthcare system

A subset of subjects did not share the same level of contentment as others.

When considering the healthcare system in Germany as a whole, none of the participants expressed strong dissatisfaction with the treatment received. However, they did express concerns about the lengthy waiting times for doctors to provide test results, as well as the need for improved patient education, which includes addressing tedious paperwork, transparent communication about alternative treatment pathways and bureaucratic processes. Most were accompanied by worry and dissatisfaction that the longer it takes for treatment to take place, the worse the upcoming result will be.

When the subjects insisted on having a certain examination that would relieve the most health concerns, they seem to have been met with dismissal or were seen as unnecessary from the respective medical staff.

*The family doctor prescribed me low-effect medication for the symptoms that I had. It was only after severely insisting, that I got the treatment that I wanted (the referral for an MRI.)* (Participant code #14)

*Since I no longer need treatment and I got healthy after the hospital visit, I am satisfied with the treatment.* (Participant code #3)

#### Negative experiences regarding the healthcare system

The responses in this subtheme revealed systemic healthcare flaws that disproportionately burdened marginalized groups, such as Albanian immigrants, while also impacting patients across Germany, albeit with varying severity depending on migration status and socioeconomic factors. Participant responses frequently cited excessive wait times for physician appointments, with several specific instances documented below.

*For my visit at the ENT (Ear, Nose, and Throat) Department there were some uncertainties with the time and place with the appointment and with the procedures. It might have also been a little bit due to the language barrier.* (Participant code #15)

*At first finding a family doctor assigned to me was difficult. I think that is because they are overloaded with patients. Also, the other thing that I am concerned about is the delayed result of the MRI.* (Participant code #14)

The treatment received was described as insufficient, since no treatment was given regarding preexisting chronic illnesses.

Albanian immigrants, who have had experience in using the healthcare system in western developed countries other than Germany, are able to make a comparison when it comes to the behavior and communication between medical staff and patients.

*I have had experiences of the healthcare system in the US as well, and there is a clear difference in the behavior of the doctors. In Germany the doctor doesn’t do any small talk. They keep everything very formal and bureaucratic. The physicians feel very unhappy with their job, and it reflects poor behavior back to the patient. In the USA they are clearly friendlier.* (Participant code #6)

#### Experiences of friends/relatives with the healthcare system and their influence on the expectations

This investigation examines the extent to which healthcare experiences within participants’ social networks shape their expectations of the healthcare system. Additionally, the study incorporates secondary accounts from social connections to broaden its analytical scope. All participants reported being influenced by the healthcare experiences of family members or others in their social circles.

Dissatisfaction due to language barriers seemed to further highlight its prominence when the study group faced the healthcare system in Germany, including doctor-patient communication and waiting time.

*A friend of mine was presented in the emergency care with appendicitis that wasn’t diagnosed properly. It subsequently led to worsening of the symptoms due to the inability to explain himself in German. After the situation worsened, the doctor showed disdain by saying that he should know how to explain himself properly in German, since he is living in Germany. But my friend was staying there temporarily and was studying in English. They influenced my expectations on the healthcare system in Germany substantially.* (Participant code #11)

There were cases where proper communication to the medical staff occurred. The lack of the language barrier enabled acute problems to get resolved properly. Participants with professional medical experience in Germany offered particularly valuable insights, identifying systemic issues that non-medical participants might overlook. Their feedback addressed critical aspects of healthcare delivery that typically fall outside patient perspectives.

*Most people would say that the waiting time at the waiting room at the clinic is a big problem. I would agree that it is a problem, as long as the problem is acute, but my wife had an acute eye problem for which she got the appropriate treatment response from the eye clinic, so I don’t see it as a big problem. Due to my work experience, I understand why the patients sometimes have to wait longer for non-acute problems. My expectation is influenced by the experience of friends and family members.* (Participant code #10)

#### Suggestions and desired changes in the healthcare system and its availability

A very prevalent suggestion was the shortening of waiting time when meeting the doctor including ER visits. Healthcare professionals capable of speaking in English or Albanian translation services were preferred, as well as a simplification of bureaucratic procedures and formalities.

*Less waiting time after going to an appointment at the doctor’s office and more personal care for the patient. Initiating small talk more often with the patient is definitely encouraged. Talking to the patient at a deeper personal level also.* (Participant code #6)

On the other hand, participants who had experience working in the medical field attributed the long waiting hours to the shortage of medical staff.

*An improvement I would like to see in the health system in Germany is more medical staff. Due to my experience as a physician, I can also say that is one of the reasons for long waiting hours for patients.* (Participant code #10)

The need to refer to unaddressed complaints following a visit was also highlighted. There is a call for allocating more time to listen to patient concerns, facilitating the ability to schedule follow-up appointments, or enabling increased communication with the physician. Small talk was also encouraged and appreciated, including talk on a deeper and personal level if the patient wishes so.

## Discussion

This qualitative study explored health literacy among 15 predominantly younger, educated Albanian immigrants in Germany, revealing that while participants demonstrated foundational knowledge of healthcare navigation and trust in physicians, significant systemic barriers impeded their access to timely and equitable care. Key challenges included language proficiency gaps that complicated communication of medical concerns and understanding of diagnoses, bureaucratic delays, prolonged waiting times for appointments, and difficulty securing family doctors. Although statutory health insurance mitigated financial concerns, systemic strains—particularly medical staff shortages—exacerbated communication gaps and care delays. Participants expressed satisfaction with treatment quality once received, yet reported inconsistent shared decision-making and occasional dismissal of their concerns by providers. These findings align with Tones’ [31] conceptualization of health literacy as not merely an individual competency but a product of systemic accessibility and institutional responsiveness. Our study reveals that even when immigrants possess foundational health knowledge, structural inefficiencies—such as bureaucratic gatekeeping and fragmented care pathways—undermine their ability to act on that knowledge, perpetuating disparities.

The health of immigrants was greatly affected by the various types of migration and the societal perceptions of immigrants [6]. Participants in this research observed that despite facing multiple health issues, such as service accessibility and comprehending healthcare procedures, the stigma tied to being an immigrant intensified their difficulties. This underscored the importance of catering to not only the physical health requirements of immigrants but also their social and mental health [5, 32]. By enhancing social support systems and fostering positive societal attitudes, health literacy and overall health outcomes for immigrant communities could be improved [12, 15]. The stressors associated with migration, such as socioeconomic instability and separation from family, could further complicate their health literacy [33, 34]. These contextual challenges were often more pronounced for immigrants compared to native populations [35]

Participants consistently highlighted bureaucratic hurdles that delayed or denied care. For instance, asylum seekers faced restrictive eligibility criteria requiring repeated documentation submissions for non-emergency treatments, while others navigated labyrinthine insurance enrollment processes tied to residency status. Such challenges resonate with Nutbeam’s [36] argument that health literacy encompasses not only personal skills but also the clarity and accessibility of health systems. Germany’s reliance on procedural rigor—such as mandatory referrals (Überweisungen) for specialist care—assumes a level of system familiarity that immigrants often lack, effectively excluding those with limited German proficiency or digital literacy.

Language proficiency surfaced as an essential element, as individuals with limited German faced increased anxiety in medical situations, which ultimately affected their capacity to adhere to healthcare directives. This was consistent with current research indicating that language served as a major barrier for immigrants, influencing both communication and understanding of medical guidance and treatment strategies [5, 23, 29, 34, 37]. Tackling these language obstacles with specific interventions, like providing multilingual services and cultural competency training for healthcare professionals [23, 38], could be vital for enhancing health results among immigrant communities.

While language proficiency was critical, structural inflexibility in healthcare protocols worsened disparities. Many medical forms, insurance documents, and digital platforms remain exclusively in German, forcing immigrants to rely on ad-hoc translations or informal networks. Nutbeam [36] emphasizes that health literacy interventions must address both functional communication (e.g., translations) and critical literacy (e.g., empowering patients to navigate systems). Yet, Germany’s institutional rigidity—such as the absence of mandated interpreter services—fails to meet these standards, exacerbating inequities.

Digitalization by offering translating services in healthcare environments could be a solution with extra environmentally friendly benefits [39]. While the study highlighted Participant #6’s emphasis on small talk as a cultural contrast between German and American healthcare norms, the limited explicit mentions of small talk across participants (only one direct example) raised questions about its universal importance in trust-building, shared decision-making, or social determinant disclosure. However, emerging evidence from broader literature [40] suggested that even subtle relational exchanges can disproportionately impact marginalized groups, warranting cautious consideration.

The persistent lack of healthcare professionals in Germany [19–21, 41–44, 44] greatly impacted the difficulties that Albanian immigrants encounter when trying to access medical services. Participants in this research, including individuals employed in healthcare, emphasized that this deficiency results in increased waiting periods for appointments and test outcomes. The scarce number of healthcare professionals limited the time that providers can spend with each patient [19–22]. This was especially clear in the stories recounted by participants who voiced their annoyance at the difficulty of locating family doctors ready to take on new patients, which frequently postponed essential specialized care. Immigrants, who often require additional time for language mediation, bore the brunt of these strains. An argument could be made that qualified medical care is not as prominent in Germany [19–22, 42–44, 44] and a full provision to fulfill the medical needs of patients who require qualified care was not fully covered [19–21]. Tackling these systemic problems was essential to guarantee that every person, especially those from marginalized communities, could maneuver through the healthcare system successfully.

While many challenges related to health literacy, such as understanding medical jargon, accessing information, and navigating the healthcare system, could be experienced by all individuals, the intensity and prevalence may vary. For example, native Germans may not face language barriers but could still struggle with health literacy due to other factors, such as education or socioeconomic status [45] and thus increase the likelihood of health problems. Certain systemic challenges, like medical staff shortages or bureaucratic complexities, affected the entire population regardless of immigration status [19, 20, 22]. Thus, while some issues were common across all groups, the specific barriers faced by immigrants were often distinct and compounded by their status.

This research highlighted the intricate relationship among health literacy, healthcare access, and the specific difficulties encountered by Albanian immigrants in Germany. Although numerous participants indicated adequate access to fundamental services, considerable obstacles persist, especially regarding language and systemic challenges like the shortage of medical personnel. These results emphasize the immediate necessity for focused actions that tackle both communication barriers [10, 29, 37, 40, 46] and the wider framework of healthcare provision [19–21, 23, 36, 38, 39, 47]. Ongoing research should keep examining these dynamics to guide effective policy measures that advance health equity.

### Limitations

The findings of this study were subject to several limitations. First, the predominance of younger (20–30 years), highly educated participants may restrict the generalizability of results to older, less educated, or socioeconomically marginalized Albanian immigrants. Second, purposive sampling via online communities risks underrepresenting isolated individuals with limited digital access. Snowball sampling further risks network homogeneity, as referrals from initial participants may over-represent similar perspectives, occupations, or residential areas. Third, all interviews were conducted in Albanian and translated into English for analysis, a process that may have introduced inaccuracies, particularly with medical terminology, despite rigorous back-translation checks. Language barriers further compounded this limitation: regional Albanian dialects and varying health literacy levels among participants could obscure nuanced expressions of health experiences. Fourth, the gender imbalance (10 men vs. 5 women) may skew findings, as cultural norms often shape gendered health-seeking behaviors and access barriers (e.g., women’s roles as family health managers), which this study may not fully capture. Finally, pre-existing researcher-participant contacts might have had unintentionally biased responses.

### Recommendations

Further investigative studies are recommended to estimate how much the effects of the medical staff shortage, influence the level of satisfaction with the medical treatment and the healthcare system in Germany as a whole. In order to get a more accurate representation of the Albanian population, further qualitative studies, with preferably more participants, are recommended.

It is suggested that a training program should be created to improve immigrants’ awareness of medical procedures and alter their attitude toward health issues. By tailoring education to their cultural backgrounds and utilizing interactive methods, this initiative could empower immigrants with the knowledge and confidence to navigate healthcare systems effectively and prioritize their well-being. Moreover, it is suggested that lengthy waiting times in the waiting room, particularly during acute emergency situations, be reduced. This could be achieved by implementing a system where new complaints or medical issues that were not adequately addressed during scheduled hospital visits are recorded and later reviewed by the attending physician. More qualitative studies on this population group are indicated for the investigation of the health literacy on a larger scale, due to the limitations of this study.

To ensure appropriate healthcare services for immigrants, it was deemed necessary to address communication barriers and improve their health literacy. Healthcare professionals should be trained to communicate effectively with immigrants, and health information should be provided in multiple languages. Additionally, efforts to improve social inclusion and reduce discrimination could contribute to better health outcomes for immigrants.

## Conclusion

This study identified several systemic deficiencies in the German healthcare system as perceived by Albanian immigrants, including prolonged waiting periods, bureaucratic hurdles, delayed test results, and challenges in accessing a primary care physician. While socioeconomic factors such as higher education and statutory health insurance helped mitigate financial concerns for some participants, systemic barriers—including communication gaps, bureaucratic delays, and provider shortages—persisted regardless of individual socioeconomic position.

Despite these challenges, participants reported positive experiences, valuing the financial protection offered by statutory health insurance and expressing general satisfaction with the quality of medical treatment. The professionalism and expertise of healthcare providers were widely appreciated, contributing to a strong sense of trust in the system even in the face of structural obstacles. This trust, coupled with the resilience demonstrated by participants, underscores both the strengths of the German healthcare system and the critical need for targeted reforms to ensure equitable access for all immigrant populations.

## Electronic supplementary material

Below is the link to the electronic supplementary material.

Supplementary Material 1

Supplementary Material 2

## Data Availability

The datasets generated and/or analyzed during the current study are available from the corresponding author on reasonable request. To protect participant anonymity, raw data will be shared only in de-identified form and after approval of a formal data access request.
