# Supplementary material for: Exploring challenges related to health literacy of Albanian immigrants in Germany: a qualitative study among a younger, educated cohort
Source: BMC Health Serv Res. 2026 Nov 5;26:1543. doi: 10.1186/s12913-026-15939-4 (PMC13741763; doi:10.1186/s12913-026-15939-4)
Supplement: Supplementary file 1 [file 12913_2026_15939_MOESM1_ESM.docx]

# Interview items / English version.

***Interview questions***

**Obtaining information on health information and services, knowledge about the functioning of the health care system in Germany**

From whom do you receive information about health, illness and treatment? How detailed is this information?

Which resources do you use to get information about health, illness and treatment?

Whom do you trust the most regarding your health?

There are different situations, in which health problems can emerge. Where do you go with different problems?

**Barriers to accessing the health care system and using health care services**

What barriers prevent you from taking advantage of health care services?

How would you evaluate the availability of health care services for you?

Imagine you/one of your family members get seriously ill. In whom would you trust the most regarding the illness and its treatment?

Do you think language and cultural differences would be a problem if you/one of your family members get seriously ill?

Are you concerned about financial issues and support in case of a serious illness?

**Past experiences with the health care system in relation to health/illness**

Which problems regarding your mental and physical health do you have?

Which part of the health care system do you seek out therefore and what kind of therapy did you receive?

Did you feel that the treatment was sufficient?

Were you sufficiently informed by the doctors about your illness and involved in the treatment decision?

**Expectations on health care system/health care workers**

What are your expectations regarding the medical staff?

How satisfied were you with situations in which you made use of the health care system?

If you have any negative experiences regarding the health care system, what happened/what was the problem?

What experiences have friends/relatives had with the health care system and do these experiences influence your own expectations (negative or positive)?

What improvements would you like to see in the health care system and its availability?
