# Supplementary material for: Exploring challenges related to health literacy of Albanian immigrants in Germany: a qualitative study among a younger, educated cohort
Source: BMC Health Serv Res. 2026 Nov 5;26:1543. doi: 10.1186/s12913-026-15939-4 (PMC13741763; doi:10.1186/s12913-026-15939-4)
Supplement: Supplementary file 2 [file 12913_2026_15939_MOESM2_ESM.docx]

# Interview items / German version.

***Interview Fragen***

**Einholen von Informationen zu Gesundheitsinformationen und -diensten, Wissen über die Funktionsweise des Gesundheitssystems in Deutschland**

Von wem erhalten Sie Informationen zu Gesundheit, Krankheit und Behandlung? Wie detailliert sind diese Informationen?

Welche Ressourcen nutzen Sie, um sich über Gesundheit, Krankheit und Behandlung zu informieren?

Wem vertrauen Sie in Bezug auf Ihre Gesundheit am meisten?

Es gibt verschiedene Situationen, in denen gesundheitliche Probleme auftreten können. Wohin gehen Sie mit verschiedenen Problemen?

**Hindernisse beim Zugang zum Gesundheitssystem und bei der Inanspruchnahme von Gesundheitsdiensten**

Welche Hürden hindern Sie daran, Gesundheitsleistungen in Anspruch zu nehmen?

Wie würden Sie die Verfügbarkeit von Gesundheitsdiensten für Sie beurteilen?

Stellen Sie sich vor, dass Sie oder eines Ihrer Familienmitglieder ernsthaft erkranken. Wem würden Sie in Bezug auf die Krankheit und die Behandlung am meisten vertrauen?

Glauben Sie, dass sprachliche und kulturelle Unterschiede ein Problem darstellen würden, wenn Sie oder eines Ihrer Familienmitglieder ernsthaft erkranken würden?

Sind Sie besorgt über finanzielle Angelegenheiten und Unterstützung im Falle einer schwerwiegenden Erkrankung?

**Bisherige Erfahrungen mit dem Gesundheitssystem in Bezug auf Gesundheit/Krankheit**

Welche Probleme bezüglich Ihrer geistigen und körperlichen Gesundheit haben Sie?

Welchen Teil des Gesundheitssystems suchen Sie deshalb auf und welche Therapie haben Sie erhalten?

Hatten Sie die Behandlung als ausreichend empfunden?

Wurden Sie von den Ärzten ausreichend über Ihre Erkrankung aufgeklärt und in die Behandlungsentscheidung einbezogen?

**Erwartungen an das Gesundheitssystem/die Beschäftigten im Gesundheitswesen**

Welche Erwartungen haben Sie an das medizinische Personal?

Wie zufrieden waren Sie mit den Situationen, in denen Sie das Gesundheitssystem in Anspruch genommen haben?

Wenn Sie negative Erfahrungen mit dem Gesundheitssystem gemacht haben, was ist passiert/was war das Problem?

Welche Erfahrungen haben Freunde/Verwandte mit dem Gesundheitssystem gemacht und beeinflussen diese Erfahrungen Ihre eigenen Erwartungen (negativ oder positiv)?

Welche Verbesserungen würden Sie sich im Gesundheitssystem und seiner Verfügbarkeit wünschen?
